# MiRNA-429 suppresses the growth of gastric cancer cells *in vitro*

**DOI:** 10.7555/JBR.26.20120029

**Published:** 2012-09-20

**Authors:** Di Liu, Peng Xia, Dongmei Diao, Yao Cheng, Hao Zhang, Dawei Yuan, Chen Huang, Chengxue Dang

**Affiliations:** a Department of Surgical Oncology, the First Affiliated Hospital, Medical School of Xi'an Jiaotong University, Xi'an, Shaanxi 710061, China;; b Department of Genetics and Molecular Biology, Medical School of Xi'an Jiaotong University/Key Laboratory of Environment and Genes Related Diseases of Ministry of Education, Xi'an, Shaanxi 710061, China.

**Keywords:** microRNA, gastric cancer, cell cycle, apoptosis

## Abstract

Micro-RNAs (miRNAs) have been found to be implicated in a very wide range of physiological processes. This study was aimed to investigate the regulation of miRNA-429 (miR-429) in gastric cancer cells on cell proliferation and apoptosis. Quantitative PCR was employed to detect the expressions of miR-429 after eukaryotic expression plasmid of miR-429 and its inhibitor were transiently transfected into poorly differentiated human gastric cancer cell line BGC823. The 3-(4,5-dimethylthiazol-2-yl)-2,5-diphenyltetrazolium bromide (MTT) reduction assays were used to examine proliferation ability. Apoptosis was analyzed by flow cytometry after transfection. The results showed that 48 h after transfection, overexpression of miR-429 reached maximum efficiency. Compared with mock transfection, miR-429 inhibited tumor cell proliferation significantly (*P* < 0.05) at 48 h and 72 h. of Overexpression of miR-429 promoted tumor cell apoptosis when compared with mock transfected cells (*P* < 0.05). On the contrary, miR-429 inhibitor promoted tumor cell proliferation and inhibited apoptosis when compared with controls (*P* < 0.05). Our results suggested that miRNA-429 may serve as a tumor suppressor during tumorigenesis of gastric cancer and may be a potential gastric cancer therapeutic target.

## INTRODUCTION

Micro-RNAs (miRNAs) are non-coding single-stranded RNA, typically containing 18-25 nucleotide sequence. It has been found that there are more than 1,000 miRNAs in the mammalian genome, and miRNAs have been found to be implicated in a very wide range of physiological processes including developmental timing, cellular proliferation, apoptosis, cell differentiation, metabolism, and hormone secretion[1]-[4]. MiRNAs are involved in tumorigenesis and development mainly through inhibiting target gene mRNA translation or degrading mRNA[5]-[7]. The cell cycle regulation system with cyclins/CDK/Rb as key molecules is an important pathway for tumorigenesis and development, among which some unknown mechanisms could play significant roles. In-depth study of miRNAs revealed that they regulate cell cycle by targeting cyclins[8],[9]. Understanding how miRNAs silence target cyclin mRNAs has been the focus of intensive research. In this study, we overexpressed miRNA449a/b in gastric cancer cells, and examined the effect of miRAN449a/b overexpression on cell proliferation and apoptosis of gastric cancer cells in details. It is believed that miR-429 could negatively regulate gastric cancer cells and potentially play a therapeutic role in the near future.

## MATERIALS AND METHODS

### Materials and reagents

Human gastric cancer cell line BGC823 was obtained from Cell Resource Center of Shanghai Institute of Biochemistry and Cell Biology, SIBS, CAS. The human gastric cancer cells were maintained in RPMI 1640 medium (Hyclone, South Logan, UT USA) supplemented with 10% fetal bovine serum (FBS) and antibiotics. Lipofectamine™ 2000 was obtained from Invitrogen (Carlsbad, CA, USA). Oligonucleotides were synthesized and purified by high-performance liquid chromatography (Augct Co. Ltd., Beijing, China). Trizol was bought from Invitrogen. RT-PCR kit was obtained from TAKARA Co. (Dalian, China).

### Construction of the eukaryotic expression plasmid pcDNA6.2-GW EmGFP-miR-429

The sequences of ha-mir-429 was obtained from miRBase (http://www.mirbase.org/). The annealed double-stranded miRNA was cloned into the linearized pcDNA6.2-GW EmGFP-miR vector. The resultant construct was transformed into *E. coli* TOP 10 competent cells. The bacteria were then plated on spectinomycin agarose LB plate and then incubated at 37°C overnight, and single colonies were chosen from the plate. The sequence was confirmed by sequencing at Beijing Genomics Institute (Beijing, China).

### Cell transfection

Cells were transfected with DNA plasmid using Lipofectamine™ 2000 (Invitrogen Grand Island, NY, USA). Logarithmically growing cells (5×10^4^ cells/L) were seeded into 6-well plate (2×10^5^ cells/well). When the cells became 70% confluent, cell transfection was conducted. Serum-free RPMI 1640 was used for rinsing twice, and 2 mL/well serum-free RPMI 1640 was added to each well. DNA-Lipofectamine™ 2000 solution was prepared according to the ratio of DNA 0.1 g : Lipofectamine™ 2000 0.5 μL. The solution was added to the following 4 groups: control, inhibitor, mock and miR-429, respectively. The medium was removed from the 6-well plate after incubation at 37°C in 5% CO_2_ for 6 h. The plate was rinsed twice with serum-free RPMI 1640. Sufficient complete medium was applied, and cells were incubated at 37°C in 5% CO_2_ for 24 h. Transfection efficiency (>90%) was confirmed by fluorescent microscopy.

### Quantitative PCR analysis

Oligonucleotides were synthesized and purified by high-performance liquid chromatography by Augct Co. Ltd.. Reverse transcription was performed using PrimeScript™RT reagent Kit. Ten µL reaction system was used at 42°C for 15 min, and at 85°C for 5 s. SYBR®Premix Ex Taq™ kit was used to perform quantitative PCR. Fifty µL reaction system was used by incubation at 95°C for 5 min, 95°C for 10 s, 60°C for 20 s, 72°C for 20 s, and 78°C for 20 s, and totally for 40 cycles. All the experiments were performed in triplicate. The expression of miR-429 was normalized against U6. Reaction data were collected.

### The 3-(4,5-dimethylthiazol-2-yl)-2,5-diphenyltetrazolium bromide (MTT) reduction assays

The cell proliferation assay was done with the MTT method. The logarithmically growing BGC823 cells (5×10^4^/L) were seeded into 96-wells plates at 100 µL/well 24 h prior to transfection. The transfections were performed as described above. Twenty µl of MTT (5 mg/mL) was added to each well and incubated for 4 h, and then the supernatant was discarded. Finally, 150 µL dimethyl sulfoxide was added to each well and oscillated for 10 min to dissolve the precipitate. Absorbance of OD was measured at 490 nm through UV spectrophotometer at 24, 48 and 72 h after transfection. The assay was performed three times in eight replicates.

### Cell apoptosis assay by flow cytometry

Cells in the logarithm stage (5×10^4^/L) were obtained and seeded (10^4^/well) into 6-well plate. When the cells reached 70% confluent, the transfections were performed as described above. Sufficient complete medium was added after 24 h. Cells were harvested 48 h post-transfection. Single cell suspension was prepared and centrifuged at 1500 rpm for 5 min. The supernatant was discarded, and cells were resuspended with PBS and then cell counting was performed. In addition, 5×10^5^ cell suspension was centrifuged at 1500 rpm for 5 min and then the supernatant was removed. Cells were washed in cold PBS, and incubated with Annexin V-FITC and propidium iodide for 15 min in the dark, and cell apoptosis was analyzed by flow cytometry within 1 h. The assay was performed three times independently.

### Statistical analysis

Statistical analysis was performed using Statistical Program for Social Sciences (SPSS) software 13.0 (SPSS Inc., Chicago, IL, USA). Independent samples t-test was used to analyze differences between groups. All experiments were done at least in triplicate and the level of significance was set at *P* < 0.05 for all tests.

## RESULTS

### The detection of miR-429 expression with real-time PCR

**Fig. 1:**
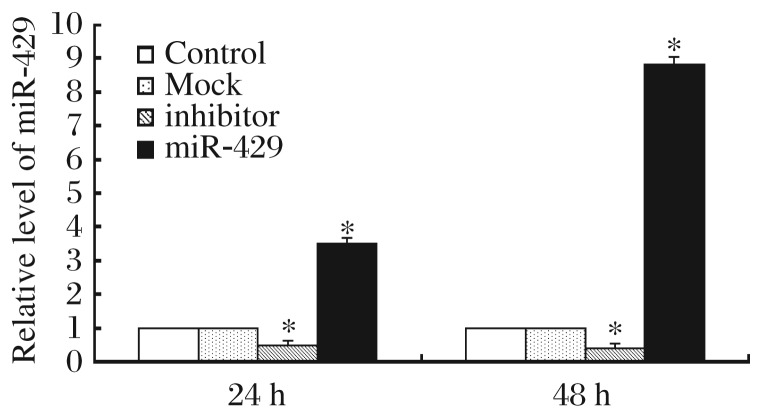
The effect of miR-429 expression plasmid and inhibitor on miR-429. Quantitative PCR was performed to detect the expression of miR-429 after transfection of human gastric cancer cells BGC823 with mock, miR-429, control or miR-429 inhibitor. Compared with control group, the expression of miR-429 was singificantly decreased in inhibitor group 24 and 48 h posttransfection (*P < 0.05). Compared with mock group, the expression of miR-429 increased significantly in miR-429 group 24 and 48 h posttransfection (*P < 0.05).

MiR-429 expression was assessed using real-time PCR after transfection with eukaryotic expression plasmid pcDNA6.2-GWEmGFP-miR-429 and its inhibitor. Total RNA was extracted from transfected cell lines by Trizol 24 and 48 h after transfection. The results showed that overexpression of miR-429 and inhibitory effect by the inhibitor reached maximum efficiency at 48 h after transfection (***Fig. 1***).

### The effect of miR-429 on the proliferation of gastric cancer cells

**Fig. 2:**
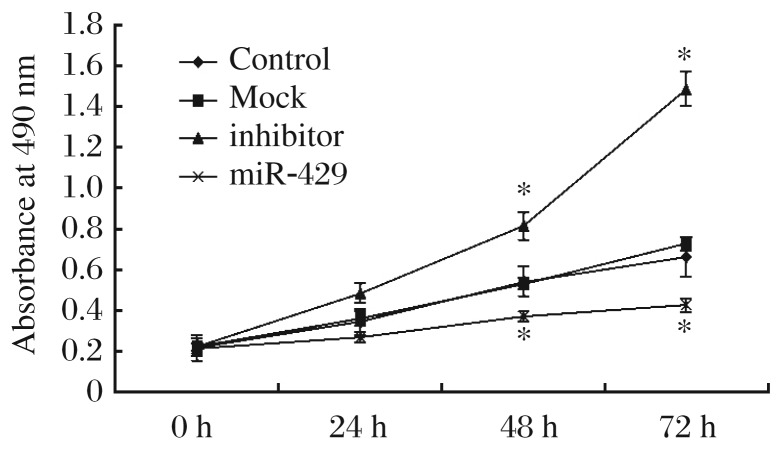
MiR-429 inhibited BGC823 cell proliferation. MTT cell proliferation assay was performed on days 1 to 3 as indicated after transfection of BGC823 cells with mock, miR-429, control, or miR-429 inhibitor.

We evaluated the functional role of miR-429 in gastric cancer cells by measuring cell proliferation in BGC823 cells which were transfected with the eukaryotic expression plasmid pcDNA6.2-GWEmGFP-miR-429 and its inhibitor. MTT assays were employed to detect the proliferation of BGC823 cell lines. Overexpression of miR-429 in BGC823 inhibited cell proliferation while downregulation of miR-429 in BGC823 cell lines promoted cell proliferation. Compared with the control group, the proliferation of tumor cells in the inhibitor groups noticeably increased at 48 h and 72 h (*P* < 0.05) (***Fig. 2***). On the other hand, compared with the mock group, the proliferation of tumor cells in the miR-429 groups were inhibited significantly at 48 h and 72 h (*P* < 0.05).

### The effect of miR-429 on apoptosis

**Fig. 3:**
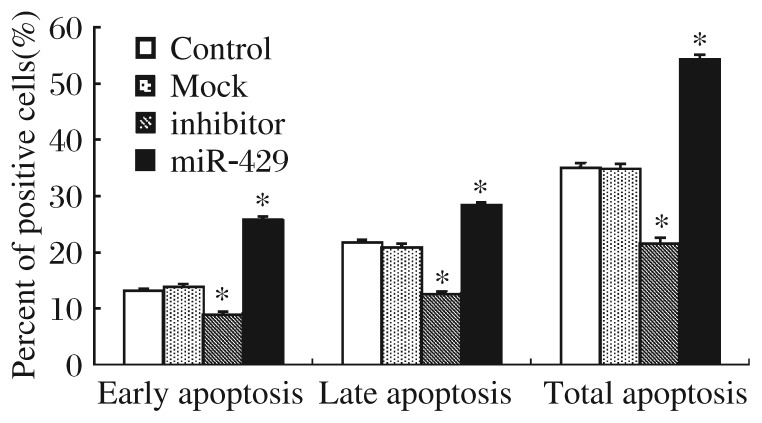
The effect of miR-429 on apoptosis. BGC823 cells were transfected with mock, miR-429, control, or miR-429 inhibitor. After 48 h, cells were collected, stained with Annexin-V and propidium iodide, and analyzed by flow cytometry. A significant difference between control and inhibitor group, mock and miR-429 group were detected in early, late apoptosis and total apoptosis (*P < 0.05)

We also explored the function of miR-429 in BGC823 cells by examining changes in apoptosis after the gastric cancer cells were transfected with the eukaryotic expression plasmid pcDNA6.2-GWEmGFP-miR-429 and its inhibitor. DNA content of transiently miRNA-transfected cells was analyzed by flow cytometry. Compared with the control group, the early and late apoptosis of BGC823 cell lines in the inhibitor group was significantly inhibited (*P* < 0.05). Compared with the mock group, tumor cell apoptosis in the miR-429 group was promoted significantly (*P* < 0.05) (***Fig. 3***).

## DISCUSSIONS

An important biological characteristic of tumors is their unrestrained growth potentiality. The activation of oncogenes and the aberrant expression of tumor suppressor genes are the main factors leading to tumors. Cell proliferation is tightly regulated by cyclins, cyclin-dependent kinase (CDK) and cyclin-dependent kinase inhibitors (CKIs). Previous studies have demonstrated that miRNAs regulated the cell cycle by targeting key proteins in signaling pathways functioning in the molecular mechanisms of tumorigenesis. An increasing number of studies have confirmed that miRNAs were involved in several cancers including cervical cancer[10], ovarian cancer[11], lung cancer[12], breast cancer[13], colon cancer[14], prostate cancer[15] and so on. This offers new ideas and inspirations in our studies on the mechanism of the effect of certain key proteins on tumor cell cycle. As a member of miR-200 family, miR-429 may be an important step in tumor progression[16]. Hu *et al*.[17] reported that downregulation of miR-200 miRNAs predicted poorer survival in ovarian cancer patients. Aberrant expression of miR-200b/200c/429 were identified in endometrial cancer[18]. MiR-200b/200c/429 were observed to contribute to cisplatin resistance by repressing AP-2α expression in endometrial cancer cells. Increasing evidence demonstrated that the miR-200 family plays a key role during tumorigenesis in some kinds of cancers. Current researches show that miRNAs regulated the expression of key proteins through combining its 5′ end and 3′-UTR of target genes[19], but this combination had poor specificity because a miRNA might have several target genes. Based on different combination rules between miRNAs and target genes, different algorithm software were designed to predict miRNAs target genes, and as the predicted target of miRNA-429, cyclins play a key role in cell cycle. Previous studies indicated that miR-26a directly targets cyclins D1 and D2 to induce cell cycle arrest in liver cancer cells[20]. Kim *et al*.[21] found that miR-93 and miR-106b inhibited P21 (a CDK inhibitor), and facilitated progression through G1 to S phase in the cell cycle. Cyclin and cyclin-dependent kinase combined to form cyclin/CDK complex, which is essential for cells from cell cycle transition. We believe that overexpression of miR-429 in gastric cancer cell lines downregulated cyclin expression by degrading target cyclin mRNA. Further work will be done to validate predicted miR-429 targets.

In this study, it was found that miR-429 had a significant effect on the biological behavior of gastric cancer cell line BGC823 after miR-429 and its inhibitor were transfected. MTT and flow cytometric studies showed that upregulation of miR-429 could inhibit cell proliferation and promote apoptosis while downregulation of miR-429 could promote cell proliferation and inhibit apoptosis. This indicated that miR-429 served as tumor suppressor in BGC823. Previous studies suggest that the miR-200 family downregulated key proteins in cell cycle post-transcriptionally by targeting its mRNA 3′-UTR region. As a result, cancer cells were arrested in G1 phase. Thereby, biological behaviors such as cell proliferation, apoptosis and so on were affected. Collectively, our results are consistent with the hypothesis that miR-429 is involved in the negative regulation of gastric cancer cells.

Recent evidence supports that miRNAs could serve as potential targets for therapy and aid in the diagnosis of disease in the future[22]. In addition, a study provided evidence that miRNAs could affect chemotherapeutic resistance of tumors[12],[23],[24]. Lawrie *et al*.[25]detected specific differential expression of miR-21 in the serum of patients with diffuse large B-cell lymphoma, which provided a basis for its potential use as a diagnosis marker, and confirmed that specific differential expression of miR-21 was closely related with patients' relapse-free survival. Similarly, the study by Si *et al*.[26] found that the proliferation and metastasis of breast cancer was prevented by lowering the expression of miR-21. Therefore, it is becoming clear that miRNAs play a key role in the tumorigenesis through different mechanisms. In this study, the clarification of miR-429 as tumor suppressor makes it possible for molecular diagnosis of miRNA-dependent tumors and miRNA-based therapies.
